# Predicting one-year post-surgical recurrence in colorectal liver metastasis using CT radiomics and machine learning

**DOI:** 10.1371/journal.pone.0330828

**Published:** 2025-08-29

**Authors:** Ningxin Chen, Boyang Zang, Yangjia Chen

**Affiliations:** 1 Department of Radiology, The Second Affiliated Hospital of Fujian Medical University, Quanzhou, Fujian, China; 2 School of Clinical Medicine, Tsinghua University, Beijing, China; 3 Department of Preventive Medicine, School of Health, Quanzhou Medical College, Fujian, China; Memorial Sloan Kettering Cancer Center, UNITED STATES OF AMERICA

## Abstract

**Background:**

Early recurrence in colorectal cancer liver metastases (CRLM) typically correlates with significantly worse survival outcomes. There is a strong demand for developing robust and interpretable approaches to assist clinicians in identifying patients at high risk of recurrence.

**Methods:**

In this study, we utilized clinical CT images and associated clinical data from 197 CRLM patients, provided as DICOM images. A total of 993 radiomic features, including shape, texture, and first-order characteristics, were extracted. Eight machine learning models were trained and validated: Random Forest (RF), Multilayer Perceptron (MLP), K-nearest Neighbors (KNN), Extremely Randomized Trees (ET), AdaBoost, Decision Trees (DT), Gradient Boosting (GB), and Linear Discriminant Analysis (LDA).

**Results:**

In predicting tumor recurrence within one year, the ET model showed the best performance using only radiomic features, with an AUC of 0.9667. RF and GB also performed well, achieving AUCs of 0.9558 and 0.9227, respectively. When combining radiomic and clinical features, the performance of all models improved in terms of AUC. Specifically, the Random Forest (RF) model achieved the highest AUC of 0.9672, followed by Gradient Boosting (GB) with an AUC of 0.9646, and Extra Trees (ET) with an AUC of 0.9459.

**Conclusion:**

We developed a CT-based machine learning model, using the Random Forest algorithm, that combines clinical (e.g., age, carcinoembryonic antigen, bilobar disease) and radiomic features (e.g., selected features included texture-based metrics such as the 90th percentile of first-order statistics in the high–low–low (HLL) wavelet-decomposed image, and Run Entropy from the gray-level run length matrix (GLRLM) in the low–low–low (LLL) sub-band.) to predict early recurrence after hepatectomy in patients with colorectal liver metastasis (CRLM). This model has the potential to guide personalized postoperative surveillance. However, limitations such as the retrospective single-center design and relatively small sample size may affect the generalizability of the findings. Further validation in larger, multi-center cohorts is warranted to confirm its clinical utility.

## 1 Introduction

Colorectal cancer (CRC) is the third most common cancer worldwide, with approximately 50% of patients developing colorectal liver metastasis (CRLM) during the course of the disease [1,2]. Hepatectomy is the optimal treatment for CRLM [3]. However, unfortunately, around 60% of patients experience recurrence within two years after complete resection of liver metastases [4]. The high recurrence rate after surgery poses a major challenge for long-term survival in CRLM patients, emphasizing the urgent need for personalized precision medicine strategies to improve patient survival and quality of life.

In recent years, radiomics of CRLM has become a valuable tool for preoperative prediction of patient diagnosis and survival after treatment [5,6]. Given the widespread use of CT in the preoperative staging of CRLM, many studies have utilized CT-based radiomic features in colorectal cancer patients for various purposes. Radiomics can help in the identification of CRLM and in detecting metachronous metastases based on microenvironmental changes in the apparently normal liver [7,8]. For example, Mühlberg et al. predicted the one-year survival of patients with metastatic colorectal cancer through geometric and radiomic analysis of CT images [9]. Similarly, Granata et al. demonstrated that contrast-enhanced MRI-based radiomics combined with machine learning analysis could predict the tumor growth front, tumor budding, and recurrence after liver resection in CRLM patients [10].

Furthermore, Deng Y. et al. identified the optimal threshold for early recurrence through statistical methods and pointed out that preoperative NLR levels, preoperative BUN levels, and TBS scores can be used to predict prognosis in patients undergoing simultaneous CRLM resection [11]. Chen Q. et al. found that elevated preoperative creatinine is an independent predictor of early recurrence in CRLM patients who underwent hepatectomy after neoadjuvant chemotherapy [12]. However, there have been relatively few studies focusing on CT-based radiomics and machine learning to predict early recurrence after hepatectomy in CRLM patients.

In this study, we developed a predictive model that combines CT radiomics and clinical features using machine learning—specifically, the Random Forest algorithm—to estimate the risk of recurrence within one year following hepatectomy in patients with colorectal liver metastasis (CRLM). This approach offers a non-invasive and quantitative method for early risk stratification, which could significantly improve postoperative surveillance and facilitate individualized patient management. A key innovation of our work is the integration of multi-dimensional radiomic features with comprehensive clinical variables within a unified machine learning framework. This combined approach allows for a more holistic characterization of tumor biology and patient status, surpassing the limitations of models that rely solely on either modality. Additionally, our study includes a comparative analysis of eight machine learning algorithms, enabling the identification of subtle imaging biomarkers often overlooked by conventional clinical assessments. Despite its potential, the study acknowledges limitations such as the retrospective, single-center design and a relatively small sample size, which may affect the generalizability of the findings. Further studies with larger, multi-center cohorts are necessary to validate the model’s clinical utility and extend its applicability to broader patient populations.

## 2 Methods

### 2.1 Patients and methods

In this study, an international institutional database from Memorial Sloan Kettering Cancer Center with waiver of informed consent was used. This international institutional database includes patients (n = 197) from 384 consecutive hepatic resections. The inclusion criteria were 1) underwent hepatectomy and histopathologically confirmed CRLM.2) surgery within 6 weeks after preoperative CT examination 3) more than 24 months of follow up and at least 90-day survival 4) complete available clinical, pathological and radiological data. Patient who received preoperative hepatic artery infusion (HAI) or local tumor ablation was excluded.

The definition of early recurrence is controversial in previous studies, and recurrence-free survival (RFS) time was previously considered as the cut-off value for early recurrence. Takahashi S et al [13] and Malik HZ et al [14] and Dai, S.et al [15] defined early recurrence as tumor relapse within 6 months after surgery for CRLM, while Imai et al. [16] determined 8 months, Liu et al. [17] reported 12 months and Chen, Q. et al [12] defined 11 months as the optimal cut-off value of early recurrence for CRLM patients, independently. In this study, we defined 12 months as early recurrence.

### 2.2 Data preprocessing

#### 2.2.1 Image cleaning.

To ensure that the machine learning model can effectively learn and accurately predict the recurrence of colorectal cancer metastasis, we applied rigorous preprocessing to the collected CT image data. The primary goal of data preprocessing is to enhance data quality, reduce the influence of noise, and create a uniform format suitable for subsequent analysis and model training.

#### 2.2.2 Image normalization.

Next, image normalization was performed to eliminate the impact of varying scan parameters on image intensity. All grayscale values were normalized to a consistent range, typically from 0 to 255. This step is critical for ensuring that the model can fairly compare and process images from different scans.

#### 2.2.3 Enhancement techniques.

To further enhance the visibility of tumor features in the images, various image enhancement techniques were applied. These included contrast enhancement, sharpening, and noise reduction algorithms. These techniques help to highlight the tumor region’s details, enabling the model to more accurately learn and identify tumor-related image features.

### 2.3 Manual annotation

Two radiologists participated in the tumor segmentation and review process. First, a radiologist with 15 years of experience manually segmented each tumor region slice by slice on the axial CT images using the ITK-SNAP software (http://www.itksnap.org/pmwiki/pmwiki.php). The tumor segmentation was performed on arterial phase images, carefully avoiding vessels and any artifacts. Then, another radiologist with 15 years of experience reviewed the segmented tumor regions to ensure accuracy and consistency.

### 2.4 Radiomic feature extraction and selection

#### 2.4.1 Feature extraction.

**Fig 1:**
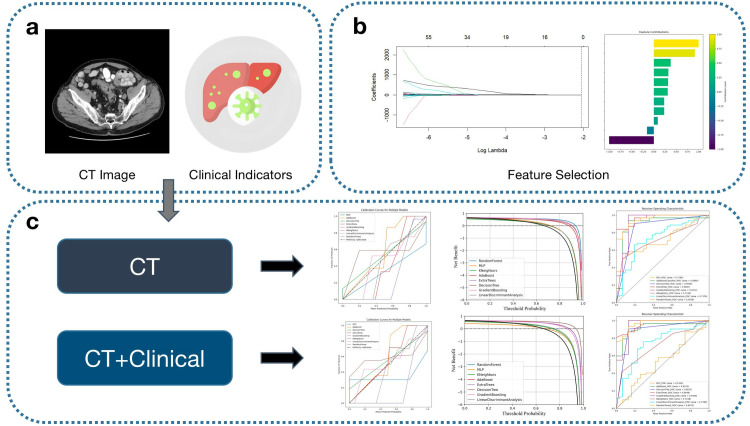
Workflow of the study. (a) Shows the types of data collected; (b) Illustrates the feature selection process; (c) Displays the results obtained from machine learning models using two different sets of features.

As shown in Fig 1, we aimed to extract reliable radiomic features from CT images of colorectal cancer metastases using the pyradiomics library, a popular medical imaging feature extraction tool. It was applied to preprocessed CT images to obtain features covering shape, texture, and signal intensity. Specifically, the extracted features included local binary pattern (LBP) features for texture description, intensity histogram features for statistical analysis, Gray-Level Difference Matrix (GLDM) features for gray level differences, texture spectrum features for texture complexity, and morphological features for shape characteristics.

#### 2.4.2 Feature selection.

The feature selection procedure encompassed several crucial steps. Initially, the Intraclass Correlation Coefficient (ICC) was employed to evaluate the stability of each feature. Those features that had an ICC value exceeding 0.75 were regarded as stable and were taken into account for subsequent analysis. Subsequently, Z-score normalization was utilized to standardize these stable features, thereby eliminating the disparities in scale and magnitude among them. Once the normalization was completed, t-tests were carried out to pinpoint the features that were significantly related to the disease status, and only those with a p-value below 0.05, which indicates statistical significance, were retained. Eventually, Lasso regression was implemented for further feature selection. Lasso regression employs L1 regularization to select features, which aids in simplifying the model and enhancing prediction accuracy. The regularization parameter of the Lasso model was optimized through cross-validation, and the most predictive features were chosen for the subsequent training of the machine learning model.

### 2.5 Predictive model for recurrence within one year

This section focuses on using eight mainstream machine learning models to predict the recurrence of colorectal cancer metastasis. Prior to model construction, all CT images underwent standardization, including voxel size resampling and intensity normalization, to ensure consistency across datasets. Radiomic features—encompassing first-order statistics, texture (GLCM, GLSZM, GLRLM), and shape features—were extracted using the PyRadiomics library. Feature selection was performed using variance thresholding and recursive feature elimination (RFE) to reduce dimensionality and eliminate redundant variables. The dataset was split into training and test sets in an 80:20 ratio, stratified by recurrence outcome. Model training and evaluation were performed using 5-fold cross-validation on the training set.

Each model was optimized and validated with predefined parameter sets as follows:

Random Forest (RF) is a potent ensemble learning algorithm, excelling in handling high-dimensional data. The n_estimators parameter is set to 100, indicating the number of decision trees generated. max_features is set to ‘sqrt’, meaning the number of features considered at each split is the square root of the total. min_samples_split is 2, and min_samples_leaf is 1, defining node splitting and leaf size thresholds.

Multilayer Perceptron (MLP) is a classic feedforward neural network suitable for complex nonlinear classification tasks. hidden_layer_sizes is set to (100,), specifying the size of the hidden layer. activation is ‘relu’; solver is ‘adam’ for weight optimization; alpha is 0.0001 as a regularization parameter; and learning_rate is ‘constant’.

K-nearest Neighbors (KNN) is a non-parametric, instance-based algorithm. n_neighbors is set to 5, weights is ‘uniform’, algorithm is ‘auto’, and p is 2, indicating Euclidean distance.

Extremely Randomized Trees (ET) is an ensemble method that constructs multiple randomized decision trees. n_estimators is set to 100, max_features is ‘auto’, min_samples_split is 2, and min_samples_leaf is 1.

AdaBoost (Adaptive Boosting) combines weak learners to form a strong classifier. n_estimators is 50, learning_rate is 1.0, and algorithm is ‘SAMME.R’.

Decision Trees (DT) split data into subsets using feature-based criteria. criterion is ‘gini’, max_depth is None (no limit), and min_samples_split is 2.

Gradient Boosting (GB) improves accuracy via iterative weak learner training. n_estimators is 100, learning_rate is 0.1, max_depth is 3, and subsample is 1.0.

Linear Discriminant Analysis (LDA) seeks optimal linear combinations of features for class separation. solver is ‘svd’, and shrinkage is None.

All models were implemented using scikit-learn in Python. Model performance was evaluated based on accuracy, precision, recall, F1-score, and area under the ROC curve (AUC).

### 2.6 Statistical analysis

In this research, we conducted an evaluation and comparison of various machine learning models with the aim of precisely predicting the metastasis and recurrence of rectal cancer. The experimental data was partitioned into a training set and a test set at a ratio of 8:2. The training set was employed for constructing the models and optimizing parameters, while the test set served to assess the ultimate performance of the models. Regarding statistical methods, this study utilized evaluation indicators such as accuracy (Acc), recall (Recall), precision (Prec), the F1 score, and the AUC value. Additionally, a confusion matrix was used to conduct a detailed analysis of the model’s performance.

## 3 Results

### 3.1 Experimental setup

All experiments were conducted on a high-performance computer equipped with a Windows 10 operating system, an Intel Core i9 processor, and an NVIDIA GeForce RTX 3080 GPU. The study was developed using the Python 3.7 programming language, primarily relying on data science and machine learning libraries such as Scikit-learn, XGBoost, Pandas, and Numpy.

### 3.2 Feature extraction and selection results

A total of 993 radiomic features were derived during this research endeavor. Out of these, 975 features were extracted from the original images, and 18 features were obtained by means of the wavelet transform method. The features extracted from the original images consist of 10 contour-related features, 20 fundamental statistical properties, 25 Gray-Level Co-occurrence Matrix (GLCM) characteristics, 18 Gray-Level Run-Length Matrix (GLRLM) elements, 14 Gray-Level Size Zone Matrix (GLSZM) aspects, and 12 Gray-Level Dependence Matrix (GLDM) details. Additionally, 130 basic statistical features, 160 GLCM elements, 135 GLRLM aspects, 120 GLSZM characteristics, and 100 GLDM details were extracted through various wavelet operations. Some of the extracted features include HLL primary MeanValue, HLL primary 60Percentile, HLL gIrlm ShortRunLowGrayLevelEmphasis, LLL gIrlm RunLengthUniformity, and gradient gldm DependenceVariance.

**Fig 2:**
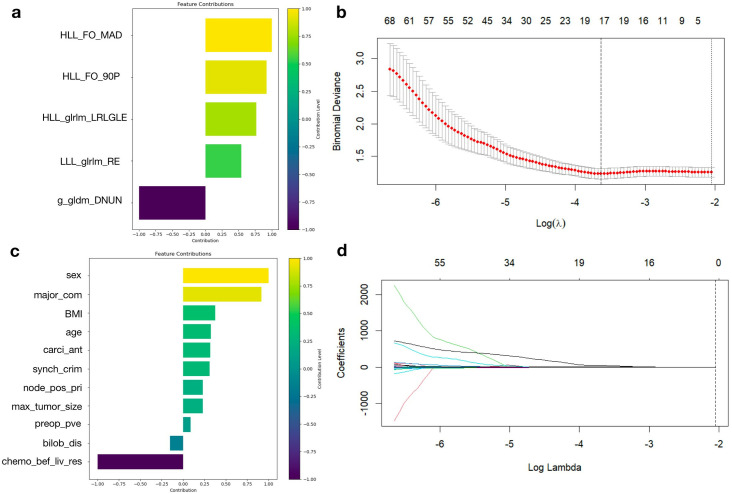
(a) Contribution of radiomic features to model performance; (b) Binomial deviance calculation for radiomic features; (c) Contribution of clinical features to model performance; (d) Changes in coefficients during LASSO regularization.

The feature selection procedure encompassed several crucial steps. Initially, the Intraclass Correlation Coefficient (ICC) was employed to evaluate the reproducibility of each feature. A total of 863 features with ICC > 0.75 were retained. These features were then standardized using Z-score normalization to eliminate differences in scale. Subsequently, univariate t-tests were performed to identify features significantly associated with recurrence status (p < 0.05), resulting in 214 features. Finally, Lasso regression with L1 regularization was applied, selecting 15 radiomic features with non-zero coefficients for model training. The selected features and their relative importance are visually presented in Fig 2a, b, d. Binomial deviance was used to assess feature-level discriminative power.

In addition, logistic regression was used to analyze the contribution of clinical indicators. All clinical features were also standardized using Z-score normalization prior to analysis. The top 10 features with the highest coefficients were retained and are shown in Fig 2c.

### 3.3 Results of the recurrence prediction models

#### 3.3.1 Imaging-based prediction models.

**Fig 3:**
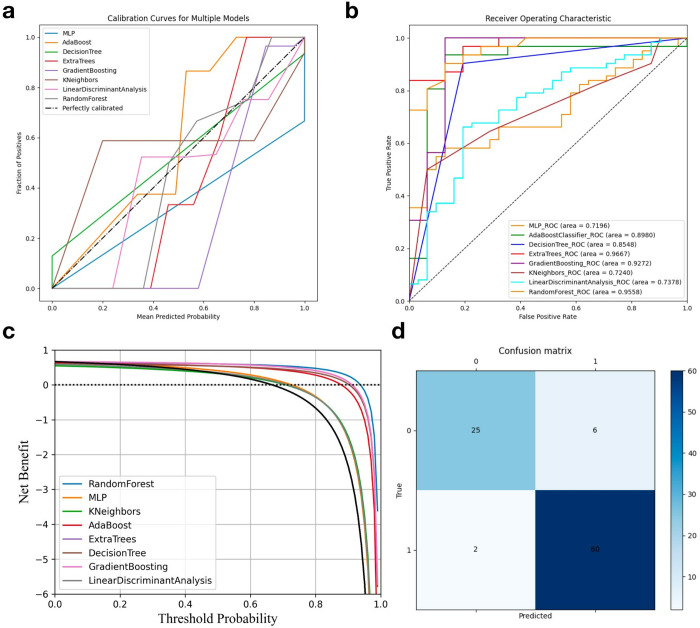
(a) Calibration curve illustrating the model’s predictive accuracy; (b) ROC curve demonstrating the model’s discriminative ability; (c) DCA curve highlighting the model’s clinical utility; (d) Confusion matrix showing the model’s performance.

**Fig 4:**
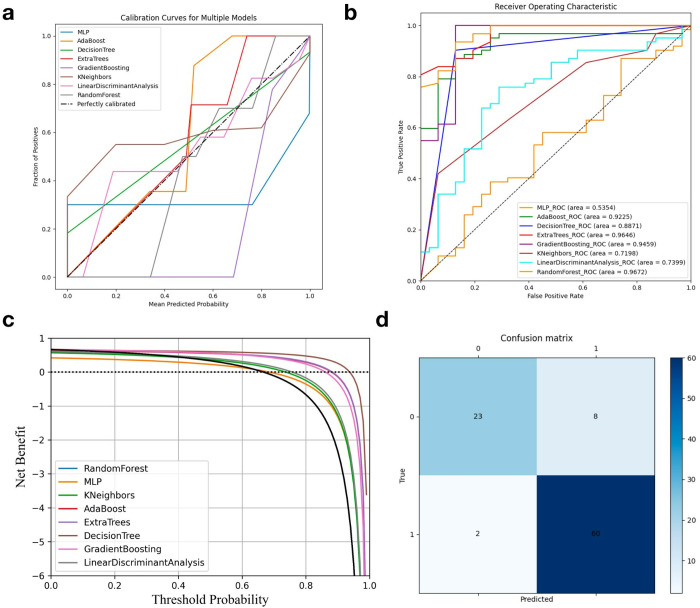
(a) Calibration curve of the final model; (b) ROC curve of the final model; (c) Comparative DCA curves of different models; (d) Confusion matrix of the final model.

The results of the imaging-based prediction models are shown in Fig 3 and Table 1. The analysis in Fig 4 demonstrates that different machine learning models exhibit varying degrees of predictive capability for recurrence. The ET model showed the highest predictive power, with an AUC of 96.67%, indicating that it accurately distinguishes between case types under all test conditions. The RF model also performed well, with an AUC of 95.58%, suggesting effective differentiation between the two tumor types in most cases. The GB and AdaBoost models also displayed strong predictive abilities, with AUCs of 92.27% and 89.80%, respectively, indicating high reliability and diagnostic accuracy. In contrast, while the LDA and KNN models performed slightly weaker, with AUCs of 73.78% and 72.40%, they still provided valuable support for recurrence prediction within acceptable ranges. Table 1 also shows the variations of each radiomics model across different metrics, with the ET algorithm achieving the highest accuracy at 0.9140.

**Table 1:** Comparison of results of machine learning prediction models based on images.

Model	ACC	PRE	REC	AUC
RF	87.10%	74.19%	85.19%	95.58%
MLP	66.67%	90.32%	50.00%	71.96%
GB	89.25%	67.74%	100.00%	92.27%
KNN	65.59%	32.26%	47.62%	72.40%
DT	87.10%	80.65%	80.65%	85.48%
AdaBoost	87.10%	74.19%	85.19%	89.80%
ET	91.40%	80.65%	92.59%	96.67%
LDA	68.82%	29.03%	56.25%	73.78%

#### 3.3.2 Imaging-clinical data fusion prediction models.

Fig 4 and Table 2 present the results of the recurrence prediction models based on fused imaging and clinical data. The machine learning models showed differences in predictive ability. When combining imaging and clinical data, the RF model performed the best, with an AUC of 96.72% and an ACC of 0.8925, demonstrating its ability to accurately distinguish cases under all test conditions. The ET model followed closely, with an AUC of 96.46%, also exhibiting excellent predictive performance. The GB and AdaBoost models showed strong predictive abilities as well, with AUCs of 94.59% and 92.25%, respectively, further proving their high reliability in diagnosis. Although the LDA and KNN models were comparatively weaker, with AUCs of 73.99% and 71.98%, respectively, they remained within a reasonable range, providing effective support for recurrence prediction.

**Table 2:** Comparison of results of machine learning prediction models based on imaging and clinical practice.

Model	Accuracy	Precision	Recall	AUC
RF	89.25%	74.19%	92.00%	96.72%
MLP	64.52%	19.35%	42.86%	53.54%
GB	93.55%	80.65%	100.00%	94.59%
KNN	69.89%	38.71%	57.14%	71.98%
DT	89.25%	87.10%	81.82%	88.71%
AdaBoost	88.17%	74.19%	88.46%	92.25%
ET	91.40%	74.19%	100.00%	96.46%
LDA	74.19%	41.94%	68.42%	73.99%

## 4 Discussion

In this study, we developed a predictive model that integrates CT radiomics features and machine learning algorithms to predict 1-year recurrence risk in patients with colorectal liver metastases (CRLM) post-surgery. The Random Forest (RF) algorithm, when applied solely to radiomics data, achieved an AUC of 95.58%, which was closely followed by the Extremely Randomized Trees (ET) model with an AUC of 96.67%. Both Gradient Boosting (GB) and AdaBoost models demonstrated strong predictive performance, with AUCs of 92.27% and 89.80%, respectively. When clinical features were integrated with radiomics data, the AUC for the RF model improved to 96.72%, while the ET model remained robust with an AUC of 96.46%. These findings underscore the exceptional stability and generalization capabilities of both the RF and ET models, particularly when dealing with high-dimensional, complex data.

The superiority of these results can be attributed to two main factors: model architecture and the nature of the prediction task. First, ensemble learning methods like RF and ET reduce the bias and variance inherent in individual models by combining the predictions from multiple decision trees. This enhances their ability to process complex, high-dimensional data. Additionally, their built-in feature importance evaluation mechanisms improve the accuracy of predictions and the interpretability of the models. Second, the risk assessment of recurrence after CRLM involves multifaceted tumor biological behavior and requires a comprehensive analysis of imaging features. Traditional imaging techniques often struggle to detect early signs of recurrence. However, radiomics analysis, through machine learning, enables the capture of subtle changes in texture, shape, and density that are critical for predicting recurrence, even when these changes are not easily identifiable in routine imaging.

Although the treatment of CRLM has been significantly improved over the past few decades due to effective chemotherapy and innovations in surgical techniques, determining which patients may benefit more from liver resection for metastatic colorectal cancer remains a major challenge for clinicians. Studies have shown that early recurrences are more aggressive and have significantly worse prognoses than late recurrences [16–18,24]. Since early recurrence of CRLM after liver resection is usually associated with significantly reduced survival, predicting early recurrence is crucial to improving patient prognosis [19,20,25]. Specifically, early identification of patients who may have early recurrence can help clinicians develop more personalized treatment strategies after surgery [21]. For example, for high-risk patients, earlier adjuvant therapy or closer follow-up may be required after surgery to detect and treat recurrent lesions in a timely manner [22,26]. In addition, such predictive models can also help screen patient groups that may benefit from more aggressive treatments, such as the addition of targeted therapy or immunotherapy, to delay the recurrence process and improve survival. In clinical practice, the application of such a predictive model can significantly optimize treatment decisions, reduce unnecessary surgeries or overtreatment, and thus improve patients’ overall treatment outcomes and quality of life [23].

In this study, we developed and validated a machine learning model that integrates radiomic and clinical features to predict early recurrence of colorectal liver metastasis (CRLM) within one year after hepatectomy. The model demonstrated high predictive performance and robustness across multiple evaluation metrics. Beyond its technical value, this model has important clinical implications. Specifically, it can serve as a non-invasive decision-support tool to stratify patients based on their recurrence risk. For high-risk individuals identified preoperatively or shortly after surgery, clinicians could consider intensifying surveillance protocols, such as scheduling more frequent imaging or follow-up visits. Additionally, these patients may benefit from tailored adjuvant therapies or inclusion in clinical trials designed to reduce recurrence risk. Conversely, patients classified as low-risk could potentially avoid unnecessary interventions, helping to reduce healthcare burden while maintaining quality care. This personalized approach aligns with precision medicine principles and may ultimately contribute to improved patient outcomes.

## 5 Conclusion

This study applied a machine learning algorithm based on CT images and clinical features to predict early recurrence within one year after liver resection in patients with CRLM. Through the extraction and selection of radiomic and clinical features from 197 patients, machine learning modeling and validation were conducted, with the Extra Trees (ET) and Random Forest (RF) models achieving the best performance (AUCs of 0.9646 and 0.9672, respectively). This method provides an effective tool for predicting early postoperative recurrence in CRLM patients, which may help optimize postoperative management and monitoring strategies. Future work will focus on validating the model in larger, multi-center cohorts and exploring its integration into real-time clinical workflows to assess its practical utility and generalizability.
